# Complications of Totally Implantable Central Venous Catheters (Ports) Inserted via the Internal Jugular Vein Under Ultrasound and Fluoroscopy Guidance in Adult Oncology Patients: A Single-Center Experience

**DOI:** 10.7759/cureus.27485

**Published:** 2022-07-30

**Authors:** Viktoria Kartsouni, Hippocrates Moschouris, Fragiskos Bersimis, George Gkeneralis, Myrsini Gkeli, Stamatia Dodoura, Aikaterini Chouchourelou, Ioannis Fezoulidis, Athanasios Kotsakis, Christos Rountas

**Affiliations:** 1 Interventional Unit of Radiology, Saint Savvas Hospital, Athens, GRC; 2 Interventional Unit of Radiology, General Hospital of Piraeus “Tzaneio”, Athens, GRC; 3 Department of Midwifery, University of West Attica, Athens, GRC; 4 Department of Radiology, University of Thessaly, Larissa, GRC; 5 Department of Oncology, University of Thessaly, Larissa, GRC

**Keywords:** parenteral nutrition, cytotoxic drugs, drug administration, chemotherapy, iv therapy

## Abstract

Introduction

In this retrospective study, the safety and complication rates of port implantations via the internal jugular vein under ultrasound and fluoroscopy guidance in adult oncology patients were analyzed.

Material and methods

Eight hundred seven ports implanted in 799 adult oncology patients at a tertiary Oncology-Anticancer Hospital during a 36-month period from January 1, 2017 to December 31, 2019 were retrospectively reviewed. Data acquisition was obtained until December 31, 2020. All procedures were performed by two specialized interventional radiologists under ultrasound and ﬂuoroscopy guidance. The vein access was via the internal jugular vein. Catheter days (the total number of days of maintenance of the port by all of the patients until removal, death, or December 31, 2020), technical success rates, and complication rates were evaluated based on the interventional radiological reports and patient medical records. Multivariate analysis regarding patients such as age, sex, body mass index (BMI), marital status, educational level, cancer type, side of insertion, diameter of internal jugular vein, diabetes, anticoagulants/antiplatelets, purpose of implantation, and catheter material as to the risk of complications was conducted.

Results

A total of 369,329 catheter maintenance days were observed (457.7±345.0). The technical success rate was 99.9%, and a total of 85 (10.5%) complications occurred, of which 24 (28.2%) occurred early (<30 days) and the remaining 61 (71.8%) were late (>30 days) complications. Specifically, 28 (3.5%) were catheter-related thrombosis (CRT), 27 (3.4%) related to infection, 17 (2.1%) were mechanical complications (16 fibrin sheath formation and one catheter occlusion), six (0.7%) related to catheter migration, four (0.5%) related to incision healing problems, and the remaining three (0.4%) related to ischemic skin necrosis. Forty-seven (5.8%) ports were removed due to complications. On multivariate analysis, cancer type was found as a risk factor for the development of a complication. Additionally, there was an indication that hematologic malignancy is related to infection.

Conclusion

Placement of ports via the internal jugular vein under ultrasound and fluoroscopy guidance is a safe procedure, with low rates of early and late complications.

## Introduction

The first placement of a totally implantable central venous catheter (port) by Niederhuber in 1982 worthily represents an important milestone in the long history of venous puncture and catheterization [1]. Since then, the use of ports has been widely established and has prevailed in the management of oncology patients [2]. Ensuring permanent venous access is critical to the frequent and prolonged administration of chemotherapeutic agents, blood products, antibiotics, contrast media, parenteral nutrition, and blood samplings [3,4]. At the same time, the safety of the patient is preserved and the quality of life is improved [5]. Hence, there is an increase in the number of ports placed internationally on an annual basis.

Although different techniques and equipment have been developed and much experience is accumulated, the implantation and presence of ports are associated with complications. Some complications are life-threatening to the patients or cause discomfort, prolong hospital stay, delay treatment, and increase hospital cost. In the modern literature, it has been documented that the radiological placement of ports is associated with high rates of technical success and low rates of complications that range from 4.4% to 14% [6]. Under this prism, a retrospective study was conducted to investigate the safety of port insertion via the internal jugular vein under ultrasound and fluoroscopy guidance and the complications of the totally implantable venous catheters in adult oncology patients.

## Materials and methods

Study population’s demographic data were recorded. In addition, medical and technical information about patients was also recorded.

Procedure

At the Interventional Radiology Department of our Anticancer and Oncology Hospital, ports were implanted in patients older than 18 years. The procedures were performed by two specialized interventional radiologists. Our equipment included an angiographic unit, Siemens Axiom Artis-Zee (Siemens Healthineers, Erlangen, Germany), and an ultrasound unit, Siemens Acuson NX3 (Siemens Healthineers, Erlangen, Germany). An eight-French (Fr) port with polyurethane Tita Jet Light II (PFM Medical, Inc., Cologne, Germany) and polyurethane Smart Port, Vortex (AngioDynamics, New York, United States), or silicone Nu port (PHS Medical GmbH, Fuldabrück, Germany) catheter was used. Upon the recommendation of the treating oncologist, a preoperative meeting was held. The patient's history and medication were recorded. Emphasis was given to anticoagulant-antiplatelet drugs, immunosuppression, previous deep vein thrombosis, reinsertion of central venous catheter, and known allergic reaction to contrast media. Recent imaging examinations and blood tests were evaluated, and an ultrasound examination of the cervical veins was performed to confirm the patency of the veins and reveal any anatomical variations or vein thrombosis. The patient was also physically examined for any anatomical or postoperation features and skin lesions on the chest. Then the patient was informed about the type and necessity of the operation as well as about any possible complications, and a written informed consent was obtained. Abnormal hemostatic function, bacteremia, or active infection that could lead to bacteremia was considered contraindications.

The procedure was performed under strict aseptic conditions and constant monitoring of the patient’s vital signs. Antibiotic prophylaxis, heparin, or sedation was not routinely used. In selected cases, 0.25-1 mg alprazolam per os (oral administration) and 1 g paracetamol intravenous (i.v.) were provided. The percutaneous approach to the right internal jugular vein was preferred because of its straight course. However, the left internal jugular vein was selected due to postmastectomy or postirradiation therapy status or when the right side was thrombosed. The chosen vein was punctured with an 18-gauge needle under ultrasound guidance. A 0.035-inch guidewire was advanced through the needle to the cavoatrial junction or inferior vena cava (ivc), under fluoroscopy guidance, using the Seldinger technique. Α micropuncture, using a 0.018-inch guidewire (Micro-Introducer Kit, (Galt Medical Corp., Garland, TX, USA)), was preferred instead when the jugular’s diameter was less than 6-7 mm, which was then exchanged through the sheath.

At the lateral thoracic area alongside and medial to the axillary skin fold and under local anesthesia, the port pocket was created. The incision’s length was about 2-3 cm, so that the chamber would precisely fit. Two absorbable sutures were passed through the pectoral fascia. The catheter was tunneled from the pocket to the puncture site and was connected to the chamber which was placed in the pocket and secured with the sutures. A nine-French peel-away sheath was then inserted over the guidewire into the vein. The catheter was twisted around the peel-away, and the correct length of the catheter was determined under fluoroscopy, so that it ended at the cavoatrial junction or 2-3 cm below in women with massive breast tissue.

The catheter was inserted through the peel-away, which was then removed. The port’s patency was confirmed by aspirating a small amount of blood and injecting a small amount of saline solution with pulsatile flow. A final fluoroscopic image documented the correct position of the catheter tip. A modified technique was employed in cases of left internal jugular vein catheterization, where the catheter was firstly placed at the cavoatrial junction and then connected to the chamber. In this setup, the catheter could be correctly guided using a hydrophilic guidewire and overcome the sharp angle at the point where the left innominate vein transits to the superior vena cava (svc). Patients remained in the hospital for 30-60 minutes, following the intervention, and were discharged having instructions about the care of the incisions. Regular visit every three to four months for the maintenance of the device regardless of its use and in cases of relevant problems was encouraged. In the department, electronic files were kept which were updated constantly. Port’s use for i.v therapy only was strongly recommended. Ports were not used systematically for blood sampling except in cases where bacteremia was investigated. For parenteral nutrition, a peripherally inserted central catheter (picc) line was inserted instead. In cases where patients had more than one complication, the most serious one was recorded in the corresponding variable.

Follow-up

A retrospective method was used for reviewing patients’ medical records between January 1, 2017 and December 31, 2020. The last date of follow-up was defined as the date December 31, 2020 or the death date by a death registry on medical records or the date of port removal. Catheter maintenance days were calculated as the number of days between implantation and the last date of follow-up.

Definition

Complications were categorized according to the Society of Interventional Radiology (SIR) classification based on the time of onset: periprocedural (<24 hours), early (<30 days), and late (>30 days) [7]. Infection could be either bloodstream infection (bacteremia, sepsis) or local infection (pocket or tunnel infection) and was diagnosed according to Infectious Diseases Society of America Guidelines (IDSA). Catheter-related vein thrombosis (CRT) was documented with ultrasound examination or venography. Fibrin sheath formation, diagnosed with venography, and catheter lumen occlusion were encountered as mechanical complications. Catheter malfunction describes the inability for proper infusion or aspiration [8]. It can be the result of different conditions such as thrombotic causes, catheter migration twisting or kinking. Thus, it was not calculated as a different category of complications, in the present study. Catheter days were calculated as the total number of days of maintenance of the port by all of the patients until removal, death, or December 31, 2020. The purpose of implantation was characterized as adjuvant, when chemotherapy was administered after tumor excision, and as nonadjuvant, when chemotherapy was administered preoperatively or in metastatic disease, in settings where cancer lesions still exist.

Statistical methods

This study’s descriptive results are presented as i) absolute frequencies with the corresponding percentages in the case of nominal or ordinal variables and ii) mean and standard deviation in the case of quantitative continuous variables. For investigating the relationship between categorical variables, Chi-Square test was used [9]. In addition, t-test was performed to determine whether the means of two data sets were different [10]. For exploring whether there are factors influencing the occurrence of a complication, binary logistic regression was performed with a variable that expresses the occurrence of complication as the dependent one and several independent variables (such as age, sex, body mass index (BMI), marital status, educational level, cancer type, side of insertion, diameter of internal jugular vein, diabetes, anticoagulants/antiplatelets, purpose of implantation, and catheter material) in order to measure their effect as risk indicators [11]. The aforementioned logistic regression, using a stepwise method, aimed to distinguish patients reporting complications for the whole sample. The corresponding odds ratios for independent variables and p-values less than 0.05 were considered statistically significant, and 95% confidence intervals (CIs) are given for odds ratios (ORs) of the aforementioned logistic regression. Data analysis was performed using the statistical software of IBM Statistical Package for the Social Sciences (SPSS, IBM Corp. Released 2017. IBM SPSS Statistics for Windows, Version 25.0. Armonk, NY: IBM Corp).

## Results

Study population: data collection

In the period between January 1, 2017 and December 31, 2019, 807 ports were implanted in 799 adult patients (530 women and 269 men) at a tertiary Oncology-Anticancer Hospital. The average age of patients was 61.6±13.1 years (the corresponding range was from 18 to 88 years), and the total number of catheter maintenance days was 369,329 (457.7±345.0 catheter days).

The average BMI corresponded to the overweight category (27.2±18.8). In all patients, port placement was indicated for the administration of chemotherapy. Study population's demographic data (sex, education level, marital status), medical information (cancer type, diabetes, anticoagulants/antiplatelets, and implantation reason), and technical information (catheter material and side of insertion) about patients are included in Table 1.

**Table 1:** Study population’s demographic and medical data ijv: internal jugular vein.

Variable	Categories	Frequency			Percent		
Sex	Male (M)	270			33.5		
	Female (F)	537			66.5		
-	-	Total	Male	Female	Total	Male	Female
Cancer type	Breast cancer	213	1	212	26.4	0.4	39.5
	Colorectal cancer	188	91	97	23.3	33.7	18.1
	Pancreatic cancer	86	40	46	10.7	14.8	8.6
	Sarcoma	68	38	30	8.4	14.1	5.6
	Lung cancer	45	25	20	5.6	9.3	3.7
	Ovarian cancer	40	0	40	5.0	-	7.4
	Gastric cancer	39	24	15	4.8	8.9	2.8
	Hematologic malignancy	6	0	6	0.7	0.0	1.1
	Other types of cancer	122	51	71	15.1	18.9	13.2
Marital status	Married	518	194	324	65.7	73.2	61.8
	Unmarried	111	42	69	14.1	15.8	13.2
	Widower/widow	74	7	67	9.4	2.6	12.8
	Divorced	86	22	64	10.9	8.6	12.2
Educational level	Primary school	274	92	182	34.6	34.7	34.5
	High school	370	122	248	46.7	46.0	46.9
	University	139	51	98	18.7	19.3	18.6
Diabetes (yes)		110	51	59	14.0	19.3	11.3
Anticoagulants/antiplatelets (yes)		150	64	86	19.0	24.2	16.4
Implantation reason	Adjuvant	274	75	199	34.7	28.4	37.8
	Nonadjuvant	516	189	327	65.3	71.6	62.2
Catheter material	Silicone	133	47	86	16.5	17.4	16.0
	Polyurethane	674	223	451	83.5	82.6	84.0
Side of insertion	Right ijv	697	261	436	86.8	96.7	81.8
	Left ijv	105	9	96	13.1	3.3	18.0

A total of 807 ports were implanted in 799 patients, i.e., in eight patients, a second port was reinserted. The technical success was achieved in 807 implantations from 808 cases that were admitted (technical success rate: 99.9%). A failure was reported in a case of svc thrombosis, where a port through the femoral vein was finally inserted. A total of 369,329 catheter maintenance days were recorded, and the mean catheter indwelling time was 457.7±345.0 days (range: 4-1,476 days). The right internal jugular vein was selected for the initial access catheterization route in 696 patients (87%). However, the left internal jugular was chosen in 104 patients (95 women and nine men). The internal jugular vein’s diameter was mostly greater than 10 mm (83.2%).

A total of 264 (32.7%) ports reached December 31, 2020 with an average number of catheter days 752.1±325.4 (range from 20 to 1,476 catheter days). From the remaining 543 (67.3%) ports, 441 (54.6%) patients did not reach the deadline due to death and 102 (12.6%) ports were removed. Among these, 54 (52.9%) ports were removed after completion of chemotherapy or in patients with no further treatment plan, 47 (46.1%) were removed due to complications, and one port was explanted upon patients’ demand. No periprocedural complication occurred. A total of 85 (10.5%) complications were observed during the follow-up period. Among them, 24 (28.2%) were early (18.1±6.8 catheter days) and the remaining 61 (71.8%) were late complications (241.6±221.1 catheter days). Bacteremia was associated mainly with sarcoma and secondarily with colorectal cancer, gastric cancer, and hematologic malignancy (Table 2).

**Table 2:** Cancer type distribution among patients with bacteremia

Cancer Type	Frequency	Percent	Cumulative Percent
Sarcoma	5	33.3	33.3
Colorectal cancer	2	13.3	46.6
Gastric cancer	2	13.3	59.9
Hematologic malignancy	2	13.3	73.2
Breast cancer	1	6.7	80.0
Pancreatic cancer	1	6.7	86.7
Other types of cancer	2	13.3	100.0
Total	15	100.0	

In addition, catheter-related thrombosis was associated mainly with colorectal cancer and breast cancer, as well as secondarily with pancreatic cancer and sarcoma (Table 3).

**Table 3:** Cancer type distribution among patients with catheter-related thrombosis (CRT)

Cancer Type	Frequency	Percent	Cumulative Percent
Colorectal cancer	8	26.7	26.7
Breast cancer	6	20.0	46.7
Pancreatic cancer	4	13.3	60.0
Sarcoma	4	13.3	73,3
Gastric cancer	3	10.0	83.3
Lung cancer	2	6.7	90.0
Other types of cancer	3	10.0	100.0
Total	30	100.0	

**Figure 1:**
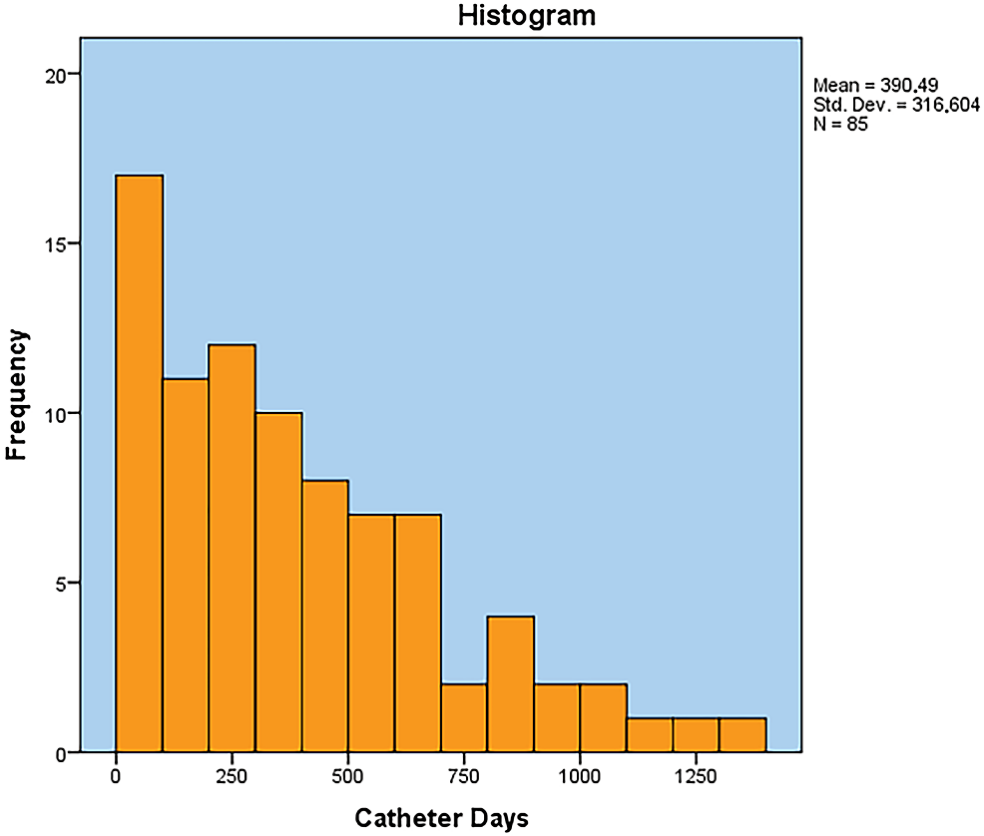
Catheter days’ histogram

It was observed that as the number of catheter days increases, the incidence of complication decreases (Figure 1). Table 4 lists the frequencies and catheter days of complications that occurred early and late, respectively, as well as the mean days from insertion to complication.

**Table 4:** Early and late complications

Type of Complication	No. of Early Complications (≤30 Days)			No. of Late Complications (>30 Days)			Total Complications			Mean Days From Insertion to Complication (Range)
	n	%	/1,000 Catheter Days	n	%	/1,000 Catheter Days	n	%	/1,000 Catheter Days	
Infection							27			
Port pocket infection	1	1.2	0.003	11	12.9	0.029	12	14.1	0.032	349.17
Blood stream infection	3	3.5	0.008	12	14.1	0.032	15	17.6	0.041	263.40
Catheter-related thrombosis (CRT)	11	12.9	0.029	17	17.6	0.046	28	33.0	0.076	106.58
Mechanical complications							17			
Fibrin sheath	4	4.7	0.011	12	14.1	0.032	16	18.8	0.043	106.12
Catheter occlusion	0	0.0	-	1	1.2	0.003	1	1.2	0.003	234.00
Catheter migration	2	2.4	0.005	4	4.7	0.011	6	7.1	0.016	94.33
Wound healing problem							4			
Pocket hematoma	1	1.2	0.003	0	0.0	0.000	1	1.2	0.003	15.00
Wound dehiscence	2	2.4	0.005	1	1.2	0.003	3	3.5	0.008	32.00
Skin necrosis	0	0.0	0.000	3	3.5	0.008	3	3.5	0.008	505.33

**Figure 2:**
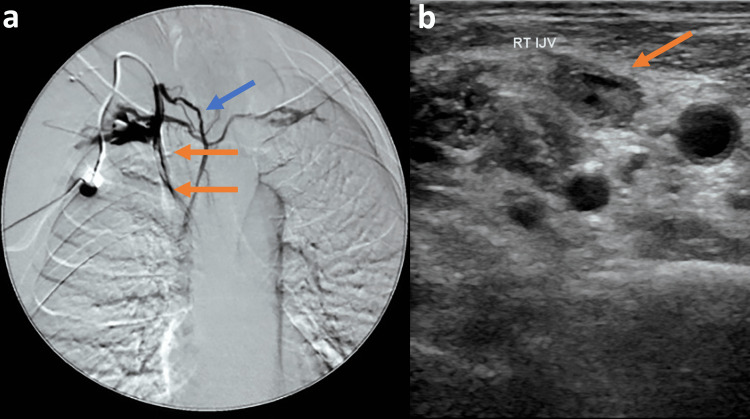
Catheter-related thrombosis a. Venography through the port, revealing right innominate vein and superior vena cava thrombosis (orange arrows), with collateral circulation (blue arrow). b. Ultrasound examination revealing right internal jugular's vein thrombosis with echogenic material in its lumen (orange arrow). RT: right, ijv: internal jugular vein.

Twenty-eight cases of vein thrombosis included 26 internal jugular vein (ijv) thrombosis and two cases of innominate and svc vein thrombosis. Eight cases were asymptomatic and revealed during the scheduled follow-up, while the remaining 22 cases of suspected vein thromboses were diagnosed with ultrasound or venography (Figures 2a, 2b).

**Figure 3:**
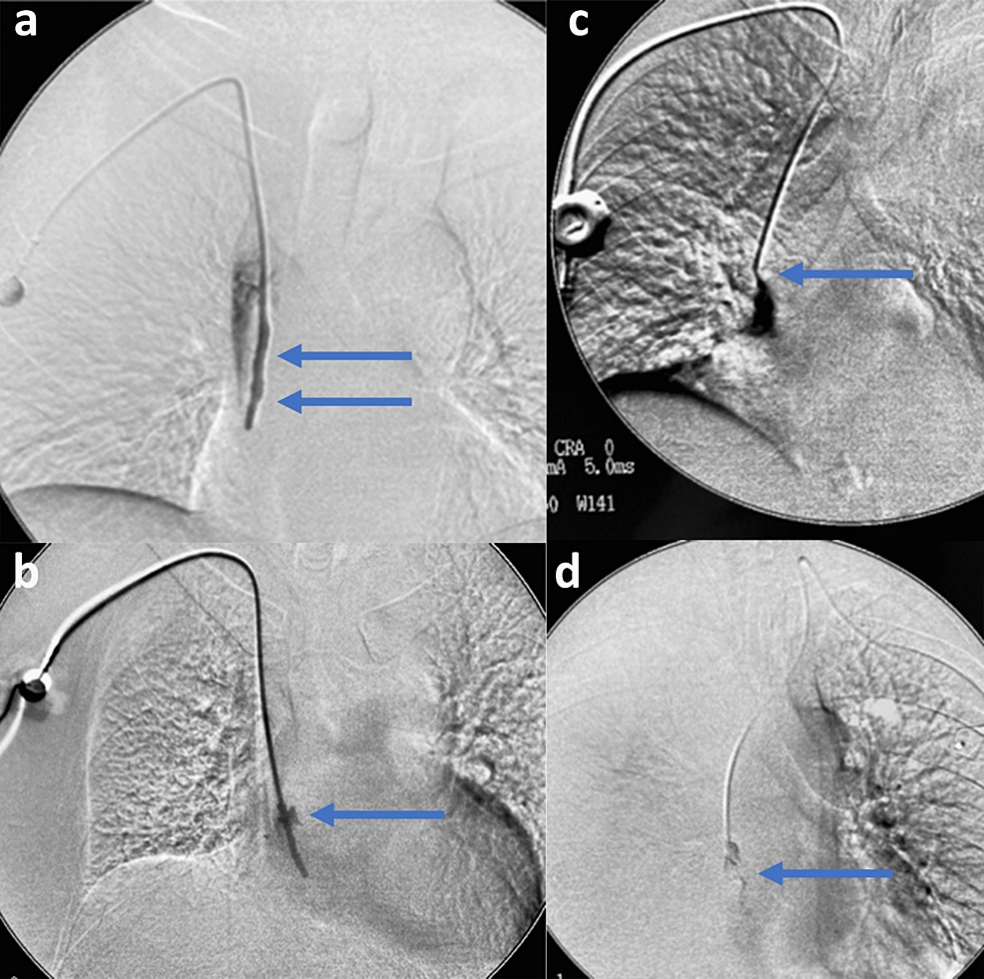
Venography through the port Characteristic images of fibrin sheath formation. a. Contrast media reflux along the proximal shaft of the catheter (blue arrows). b. Contrast media leakage through side halls of fibrin sheath (blue arrow). c. Contrast media fills a fibrin sheath extending the tip of the catheter, causing a wind sock appearance ((blue arrow). d. Contrast media filling defect at catheter tip (blue arrow).

Anticoagulants were prescribed, while none of the ports was removed due to vein thrombosis. The 16 cases of fibrin sheath formation, suspected of catheter malfunction, were documented with venography (Figures 3a-3d).

**Figure 4:**
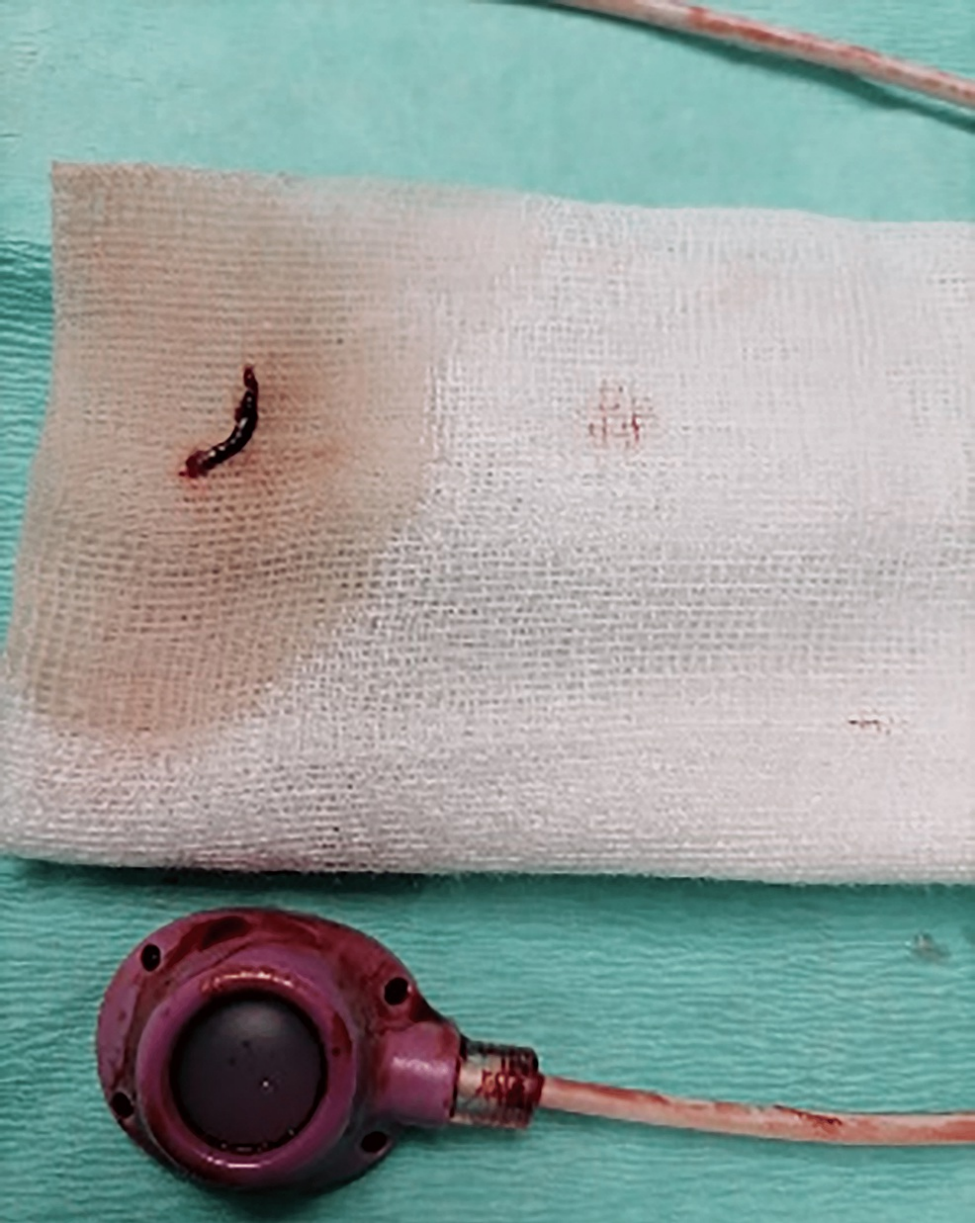
Removal of a totally occluded catheter due to an intraluminal clot

Two cases of fibrin sheath formation were successfully treated with catheter stripping. Nine cases had to be removed, while in five cases, ports were used under precaution. The totally occluded catheter was removed (Figure 4).

**Figure 5:**
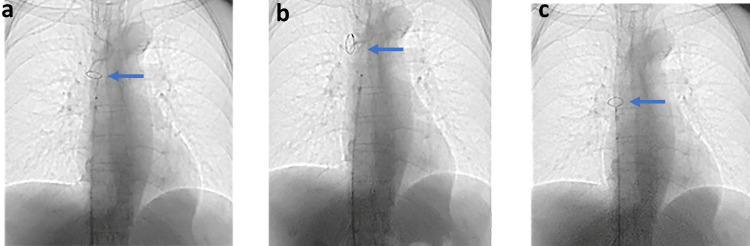
Catheter migration, nine months after port insertion a. A snare catheter (blue arrow) was inserted through the right common femoral vein, b. the tip of the catheter (blue arrow) was captured by the snare, and c. then pulled down, restoring the catheter’s position and patency (blue arrow).

The 27 cases of infection included 15 cases of bacteremia and 12 cases of local infection in the pocket. The pathogen microorganisms in bacteremia were Gram-positive cocci in eight cases, Gram-negative cocci in five cases, and fungus in two cases. The most common Gram-positive cocci was *Staphylococcus* spp. especially *Staphylococcus epidermidis*, while most often Gram-negative cocci was *Enterobacter* and *Pseudomonas aeruginosa*. *Candida* spp. was isolated in fungus category. Twenty-six ports were removed due to infection, while one case with local infection regressed with antibiotic therapy. One case of catheter migration was corrected with a snare catheter (Figures 5a-5c). The other five cases had to be removed. Three ports were removed due to healing problems and three due to skin necrosis.

In the present study, five out of six patients with hematologic malignancy had complications. Three cases were categorized as infection, one as catheter-related thrombosis, and the last one as healing problem. Therefore, there is an indication that hematologic malignancy could be associated with infection. In addition, catheter-related thrombosis was not associated with anticoagulants (χ^2^=0.653 p=0.634) or placement reason (χ^2^=3.229, p=0.080). Comorbidity of diabetes was not associated with catheter-related thrombosis (χ^2^=0.011 p=0.999) and bacteremia (χ^2^=0.680, p=0.708), and age <65 years was not associated with the occurrence of complication (χ^2^=3.025, p=0.085). The catheter’s material did not affect the appearance of catheter-related thrombosis (χ^2^=0.951, p=0.454) or the appearance of infection (χ^2^=0.056, p=0.999). In addition, no correlation was observed between the occurrence of catheter-related thrombosis and the diameter of the jugular vein (χ^2^=0.386, p=0.463). The movement of the catheter was not related to the body mass index (t=0.356, p=0.722). The average number of catheter days for patients with placement reason "adjuvant" (589.10) was statistically significantly higher than the average number of catheter days for patients with placement reason "nonadjuvant " (373.99) (t=8.291, p<0.01).

Binary logistic regression (BLR) was conducted with “occurrence of complication” as the dependent variable (Table 5). For the whole sample, the only independent variable reﬂecting factors that affect the occurrence of complication was cancer type (reference category: sarcoma, rest categories: breast cancer, pancreatic cancer, colorectal cancer, gastric cancer, lung cancer, ovarian cancer, hematologic malignancy, other types of cancer). Specifically, patients with breast cancer (OR=0.487, p=0.060), pancreatic cancer (OR=0.317, p=0.028), colorectal cancer (OR=0.421, p=0.030), and lung cancer (OR=0.197, p=0.039) were associated with a reduced probability of complication’s occurrence compared to sarcoma. On the contrary, patients with hematologic malignancy (OR=21.154, p=0.007) were associated with a higher probability of complication’s occurrence compared to sarcoma. The selected BLR model is considered statistically significant (χ^2^=28.814, p<0.01) with a relatively satisfactory predictive accuracy (90.0%) (Table 6).

**Table 5:** Variables in the BLR’s equation BLR: binary logistic regression, df: degrees of freedom, Sig: significance level (p-value), B: beta coefficient in logistic regression model.

Explanatory Variables	B	SE	Wald	df	Sig.	Exp(B)	95% CI for EXP(B)
Sarcoma			23.212	8	0.003		
Breast cancer	-0.719	0.382	3.544	1	0.060	0.487	(0.231, 1.030)
Pancreatic cancer	-1.148	0.524	4.804	1	0.028	0.317	(0.114, 0.886)
Colorectal cancer	-0.866	0.400	4.695	1	0.030	0.421	(0.192, 0.921)
Gastric cancer	-0.077	0.519	0.022	1	0.881	0.925	(0.335, 2.559)
Lung cancer	-1.626	0.786	4.274	1	0.039	0.197	(0.042, 0.919)
Ovarian cancer	-1.070	0.675	2.513	1	0.113	0.343	(0.091, 1.288)
Hematologic malignancy	3.052	1.138	7.191	1	0.007	21.154	(2.274, 196.825)
Other types of cancer	-0.974	0.452	4.645	1	0.031	0.378	(0.156, 0.916)
Constant	-1.442	0.308	21.876	1	<0.001	0.236	

**Table 6:** BLR’s evaluation criteria BLR: binary logistic regression.

Sample	Omnibus Tests of Model Coefficients		Model Summary			Classification Table (Cut Value=0.50)
	Chi-Square	p-value	-2 Log Likelihood	Cox and Snell R Square	Nagelkerke R Square	Accuracy
Total	28.814	<0.01	514.515	0.035	0.072	90.0%

## Discussion

It is documented in the literature that the placement of a port under ultrasound and fluoroscopy guidance using the Seldinger technique ensures low complication rates and high technical success rates [12,13]. The Seldinger technique when performed under ultrasound guidance has almost 100% success even from the first attempt, avoiding the possibility of hematoma and trauma that increases the chance for thromboses [14,15]. It is also associated with a total complication rate of 6.6%-14%, while for anatomical landmark-guided surgical insertion, the reported complication rates range from 5% to 24.6% [16]. On the other hand, the complication rates of the cut-down technique via the cephalic vein range from 16% to 21% [17]. The internal jugular vein (ijv) is considered superior to subclavian vein (scv) catheterization regarding the technical success, complication rates, and procedure time, due to its direct visualization, its distance from the lung apex, and its straight course, concerning the right side [18]. Some studies support that ijv catheterization carries a lower incidence of thrombosis than scv catheterization [19,20]. In a meta-analysis by Wu et al., the authors identified the superiority of the ijv regarding the incidence of major mechanical complications, but they did not reveal any statistically significant difference in infections and thrombotic complications [21]. At the same time, the fluoroscopy guidance is necessary not only to verify the correct length of the catheter but also to correct any unsuitable course of the guidewire or the catheter. The overall complication rate of radiological placement of a port has been reported in a study from 4.4% to 14% [6].

In our study, the technical success rate was 99.9%, while no periprocedural complication was recorded. The overall complication rate was 10.5% (incidence: 0.23 complications/1,000 catheter days). Teichgraber et al. also reported 99.8% technical success, while total complication rate was 11.8%, (incidence: 0.41 complications/1,000 catheter days) in 3,160 port implantations [22], while Maureau et al. reported total complication incidence 0.52%/1,000 catheter days [23]. 

Also, cancer type was found as a risk factor for the development of a complication, while hematologic malignancy and sarcoma were associated with high rates of complications. Several other studies have identified that certain cancer types, such as pancreatic and gastric cancers [24], hematologic malignancy [25], or breast and lung cancer [26], are associated with an increased risk of complications. Regarding the distribution of complications in correlation to catheter days, it was observed that as the number of catheter days increases, the incidence of complication decreases, but no clear threshold was documented, although Voog et al. reported that complications were extremely rare after one year [27]. Although the number of catheter days for patients with adjuvant placement (589.10) was higher than the number of catheter days for patients with nonadjuvant placement (373.99), surprisingly the first group, having zero tumor burden at the time of insertion, had more complications. We hypothesize that other factors interfere like cancer type and performance status.

Catheter-related thrombosis rates were 3.5% (incidence: 0.076/1,000 catheter days). Catheter-associated thrombosis has a reported incidence of 0.3%-28.3% [28]. Luciani et al. observed that the upper extremity catheter-related vein thrombosis is not a rare occurrence (11.7%) and is usually asymptomatic [29]. In a large series of 51,049 patients, 1.8% developed an upper extremity thrombosis [30], and in a systematic review where the incidence of symptomatic vein thromboses varied from 0.3% to 28.3%, the incidence of venography (mostly asymptomatic) ranged from 27% to 66% [31]. Our numbers of vein thrombosis include some asymptomatic cases (8/28) that were revealed during the scheduled follow-up. Nevertheless, no patients needed hospitalization or removal of the device and all of them were treated with heparin. Fibrin sheath formation rate was 1.98% (incidence: 0.044/1,000 catheter days). It represents a common problem that can be restored with flushing, thrombolysis, or catheter stripping, but it may also lead to port removal. Anticoagulants did not affect the occurrence of vein thrombosis or fibrin sheath formation, and this is in accordance with the clinical practice guidelines, where routine prophylaxis with anticoagulants to prevent thrombotic events is not recommended [32,33]. Risk factors such as vessel diameter (<10 mm), left jugular vein catheterization, obesity, age, ovarian cancer, and diabetes were not documented in our study. Also, right jugular vein catheterization was not associated with lower rates of vein thrombosis.

Infection rate was 3.4% (incidence: 0.073/1,000 catheter days) which was the most serious complication leading to prolonged hospitalization in some cases and removal of the device. Incidence of port-associated infection ranges from 0.6% to 27% [34]. Most guidelines recommend 0.3 infections/1,000 catheter days as an appropriate upper threshold for port implantation [35]. Few studies have shown that hematogenous malignancy is better associated with infections than solid tumors [36-38], a fact that is observed as well in our study, although the number of cases is relatively small. Impaired patient immunity may be responsible for the development of this complication. There was no association between infection and diabetes, a fact that is supported by other investigators [39,40]. The most common microorganism identified on blood and/or catheter cultures was *Staphylococcus epidermidis*, followed by *Enterobacter*, *Pseudomonas aeruginosa*, and *Candida* spp. The same results are reported in several studies [41,42]. These microorganisms which are isolated on the skin, gastrointestinal tract, urinary system, and environment can contaminate the port and lead to infection. It is very crucial for the aseptic technique to be applied meticulously not only during the implantation but also during the incision’s healing and in every use.

In our study, 25 ports out of 807 (3%) were removed due to infection, which reaches the percentage reported in the study of Shim et al., where 45 out of 1,747 (2.6%) implanted ports were explanted due to infection [43]. Vidal et al. reported that 81% of infections required port removal, while conservative treatment and port salvage were feasible only in a few cases [44]. It is documented in the literature that infection is the most frequent indication for port removal [5]. The catheter material did not affect the appearance of thrombosis or infection. Some investigators report that some types of polyurethane may be associated with a higher incidence of thrombosis, while silicone may be associated with better biocompatibility [45]. Wildgruber et al. concluded that polyurethane catheters are more susceptible to catheter-related infections and exhibited a higher thrombogenicity, compared to silicone catheters [46]. Busch et al. reported more thrombotic catheter occlusions in silicone catheters and more venous thromboses in polyurethane catheters [47]. However, Biffi et al. reported that there is no specific recommendation regarding materials for clinical practice [48].

Catheter migration can lead to cardiovascular, neurologic, thrombotic, and infectious complications [49]. The increase of intrathoracic pressure due to coughing, sneezing, weight lifting, changes in body position, or physical movements and a high infusion flow rate are considered as possible mechanisms [50]. It is restricted when the procedure is performed under fluoroscopy guidance and the catheter tip is located at the cavoatrial junction or in the proximal right atrium. It is not clear in the literature whether catheter migration is correlated with BMI [51-53]. In our study, where six cases (0.7%, incidence: 0.016/1,000 catheter days) were recorded in women only, no association was revealed. It is important that catheter migration may be corrected with a snare catheter; otherwise, it has to be removed. Healing problem rate was 0.4% (incidence: 0.011/1,000 catheter days). Pocket hematoma occurs with an incidence ranging between 0% and 4.5% [54]. A small hematoma is a relatively common event which gradually resorbs, but a severe hematoma needs drainage and may lead to port removal. It is reported that intravenous heparin initiation six hours or 24 hours after pocket creation is associated with 20% prevalence of pocket hematoma formation, while warfarin therapy or no anticoagulation is associated with only 2%-4% risk of pocket hematoma formation [55]. Wound dehiscence may be restored, although there is a high probability of local infection. Anticoagulants did not affect the probability of hematoma or diabetes in the occurrence of wound dehiscence.

Skin necrosis rate was 0.4% (incidence: 0.008/1,000 catheter days) and occurred in patients who had severe weight loss. In the study of Kim et al., the reported rate was 0.7% [28]. It is a relatively rare complication which also leads to port removal. Risk factors include BMI, extravasation, metastatic carcinoma, and local infection [56]. The malfunction was not encountered as a specific category in our study, as it is the result of thrombotic complications or migration. It is true that the definition of complications varies and the calculation of rates differs, and this provokes confusion in the literature and difficulty to compare complication rates.

The frequency of port handling and whether or not aseptic technique is applied in every use are important factors that cannot be calculated. Careful preoperative assessment, port implantation by interventional radiologists, and constant application of strict sterile conditions are important factors for the prevention of the most common complications. Moreover, interventional radiology methods comparing to surgical methods have the advantage to guide and restore in real time the route and position of the catheter. Also, some complications such as catheter migration and fibrin sheath formation cannot be corrected surgically. Interventional radiology methods achieve these corrections easily and bloodlessly.

Limitations

Our study was retrospective in a single institute, without control group. The low incidence of complications did not allow us to identify other potential prognostic factors. Nevertheless, it was a detailed analysis that confirmed the safety of the radiological placement of ports.

## Conclusions

Placement of ports via the internal jugular vein under ultrasound and fluoroscopy guidance is a safe procedure, with low rates of early and late complications. Interventional radiologists eliminate the immediate complications and manage successfully the late complications such as fibrin sheath formation and catheter migration, salvaging the port. Cancer type is a risk factor for the development of a complication. Hematologic malignancy and sarcoma are correlated with high rates of complications. Additionally, hematologic malignancy may predispose to infection. A standardized classification of complications has to be established.
